# Genome Sequencing of Fiber Flax Cultivar Atlant Using Oxford Nanopore and Illumina Platforms

**DOI:** 10.3389/fgene.2020.590282

**Published:** 2021-01-14

**Authors:** Alexey A. Dmitriev, Elena N. Pushkova, Roman O. Novakovskiy, Artemy D. Beniaminov, Tatiana A. Rozhmina, Alexander A. Zhuchenko, Nadezhda L. Bolsheva, Olga V. Muravenko, Liubov V. Povkhova, Ekaterina M. Dvorianinova, Parfait Kezimana, Anastasiya V. Snezhkina, Anna V. Kudryavtseva, George S. Krasnov, Nataliya V. Melnikova

**Affiliations:** ^1^Engelhardt Institute of Molecular Biology, Russian Academy of Sciences, Moscow, Russia; ^2^Federal Research Center for Bast Fiber Crops, Torzhok, Russia; ^3^All-Russian Horticultural Institute for Breeding, Agrotechnology and Nursery, Moscow, Russia; ^4^Moscow Institute of Physics and Technology, Moscow, Russia; ^5^Peoples' Friendship University of Russia (RUDN University), Moscow, Russia

**Keywords:** flax, *Linum usitatissimum* L., *de novo* genome assembly, Nanopore, Illumina, pure high-molecular-weight DNA

## Introduction

Flax (*Linum usitatissimum* L.) has been grown for seeds and fiber since ancient times (Vaisey-Genser and Morris, 2003). Fiber flax is taller than linseed and has branches only in the upper part of the stem. Linseed branches begin from the middle part of the stem, and these plants produce many large seeds (Diederichsen and Richards, 2003). Flax seeds are rich in omega−3 fatty acids and lignans, the health benefits of which have been proven in numerous studies (Caligiuri et al., 2014; Goyal et al., 2014; Kezimana et al., 2018; Parikh et al., 2019). Therefore, linseed is used in the food and pharmaceutical industries, animal feeds, and the production of eco-friendly paints and composites (Singh et al., 2011; Corino et al., 2014; Goyal et al., 2014; Campos et al., 2019; Fombuena et al., 2019). Flax fibers are hollow tubes that mainly consist of cellulose; they have high strength and durability, which allows one to use them in the production of high-quality textiles (Vaisey-Genser and Morris, 2003). Flax fiber has a high absorbent capacity owing to the wicking and movement of moisture along the surface, enabling its use in cloth for hot climates, sails, tents, and rugs (Atton, 1989). However, it is possible to obtain a long fiber only from a part of the flax stem with no branches; therefore, despite high quality, linen fibers have to a large extent been displaced by synthetic fibers (Muir and Westcott, 2003). Nevertheless, awareness of ecological problems has attracted attention to the use of materials that are more sustainable for our planet, and interest in flax fibers is reviving. Additionally, in the last few years, flax fiber has been actively used as a component of composite materials with good potential for automotive, aerospace, and packaging applications in which high fiber length is not very important (Zhu et al., 2013; Mokhothu and John, 2015; Wu et al., 2016; Dhakal and Sain, 2019; Fombuena et al., 2019; Goudenhooft et al., 2019; Zhang et al., 2020a).

The genome of linseed cultivar CDC Bethune was sequenced on an Illumina platform in 2012, using paired-end and mate-pair libraries. This resulted in an assembly of 302 Mb with scaffold N50 of about 700 kb, contig N50 of ~20 kb, and 81% coverage of the flax genome estimated at 370 Mb (Wang et al., 2012). Chromosome-level assembly for 15 chromosome pairs of CDC Bethune was obtained in 2018, using BioNano genome optical, BAC-based physical, and genetic mapping (You et al., 2018). Scaffold-level genome assemblies of linseed cultivar Longya-10, fiber cultivar Heiya-14, and pale flax were generated in 2020, based on Illumina sequencing, Hi-C technology, and genetic mapping (Zhang et al., 2020b). These results are extremely important for further progress in molecular studies of flax, the development of genome editing, and marker-assisted and genomic selection (Saha et al., 2019; Morello et al., 2020; You and Cloutier, 2020). A high-quality genome can be used as a reference for genome and transcriptome assemblies of different flax cultivars/lines, and the identification of polymorphisms and differences in gene expression within *L. usitatissimum* genotypes (Dmitriev et al., 2019, 2020b; Guo et al., 2019; Wu et al., 2019). Genome sequences of flax are necessary for the identification of particular gene families or repeat classes in species of the genus *Linum* and cultivars/lines of *L. usitatissimum* (Bolsheva et al., 2019; Novakovskiy et al., 2019; Ali et al., 2020; Dmitriev et al., 2020a).

Recent studies have shown that different genotypes of the same crop can diverge greatly at the genome level, not only in terms of SNPs and small indels but also long insertions and deletions, which can be identified by comparing high-quality genome assemblies (Zhang et al., 2019; Song et al., 2020). Next-generation sequencing platforms, such as Illumina, SOLiD, 454, Ion Torrent, and BGISEQ, have enabled the determination of genomic sequences for thousands of plant genotypes using short reads, whereas the development of third-generation sequencing platforms, such as Pacific Biosciences (PacBio) and Oxford Nanopore Technologies (ONT), which produce long reads of up to hundreds of thousands of bases, has facilitated accurate genome assembly (Goodwin et al., 2016; Li et al., 2017; Belser et al., 2018; Li and Harkess, 2018). Despite the wide use of third-generation sequencing approaches in studies of plant genomes, we did not find such sequencing data for flax. To fill this gap, we sequenced the genome of fiber flax cultivar Atlant using ONT and Illumina platforms to obtain a combination of long reads with insufficient accuracy and short high-precision reads, which is extremely important for high-quality genome assembly.

## Materials and Methods

### Plant Material

Fiber flax cultivar Atlant (alias—l. 23-4 Saldo × Mogilevskij) is characterized by high values of parameters that determine the quality of fiber, including flexibility, metric number, linear density, and calculated relative breaking load. Additionally, this cultivar has low variability of morphological and anatomical characteristics under stress conditions, especially unfavorable soil pH, compared to optimal ones (Ryzhov et al., 2012; Rozhmina et al., 2020). These characteristics of cultivar Atlant are important for the guaranteed production of high-quality fibers that meet the requirements of the textile industry.

Atlant seeds were obtained from the Institute for Flax (Torzhok, Russia), which is the originator of this cultivar. Seeds were sterilized in 1% sodium hypochlorite for 2 min and planted in 20 cm pots with sterile soil. Plants were grown in a climate chamber (Daihan LabTech, South Korea) for 2 weeks, and then leaves were collected from individual plants, frozen in liquid nitrogen, and stored at −80°C until DNA extraction.

### DNA Extraction

The DNA extraction method included the homogenization of 0.1 g of leaves from a single plant in liquid nitrogen followed by DNA isolation using a DNA-EXTRAN-3 kit (Synthol, Russia), DNA precipitation with CTAB-containing buffer (1% CTAB, 50 mM Tris-HCl pH 8.0, and 10 mM EDTA), and purification in ion-exchange columns from the Blood and Cell Culture DNA Mini Kit (Qiagen, USA). The DNA concentration and quality were evaluated using a Qubit 2.0 fluorometer (Life Technologies, USA) and NanoDrop 2000C spectrophotometer (Thermo Fisher Scientific, USA). The DNA length and control of RNA absence were assessed *via* electrophoresis using a 0.8% agarose gel.

### Genome Sequencing on ONT Platform

Library preparation was performed using an SQK-LSK109 Ligation Sequencing Kit (ONT, UK) for 1D genomic DNA sequencing. Minor modifications were introduced to the basic protocol for library preparation. The incubation time was increased to 20 min at the DNA recovery step and 60 min at the adaptor ligation step. A MinION (ONT) instrument with an R9.4.1 flow-cell (ONT) was used for sequencing.

### Genome Sequencing on Illumina Platform

DNA was fragmented on an S220 ultrasonic homogenizer (Covaris, USA), and 1 μg of fragmented DNA was used for library preparation using a NEBNext Ultra II DNA Library Prep Kit for Illumina (New England Biolabs, UK) with a size selection of adaptor-ligated DNA of ~600–800 bp. The DNA library concentration and quality were evaluated on a Qubit 2.0 fluorometer (Life Technologies) and 2100 Bioanalyzer (Agilent Technologies, USA), respectively. Sequencing was performed on a HiSeq 2500 instrument (Illumina, USA) with a read length of 250 + 250 bp.

### Preliminary Data Analysis

For successful Nanopore sequencing, DNA quality is crucial. We developed a protocol for the isolation of long high-purity DNA from a single flax plant and obtained DNA of ~50 kb with A260/A280 of 1.9 and A260/A230 of 2.0. The DNA concentrations measured with a NanoDrop spectrophotometer (Thermo Fisher Scientific) and Qubit fluorometer (Life Technologies) had similar values, which is an important criterion of DNA purity. The sequencing of the obtained DNA on the ONT platform produced 8.4 Gb with N50 of 12 kb, corresponding to ~23 × flax genome coverage. On the Illumina platform, 30 × genome coverage was obtained with 22.6 million 250 + 250 paired-end reads. The raw data were deposited in the NCBI Sequence Read Archive (SRA) under the BioProject accession number PRJNA648016.

First, the MinION fast5 files were processed using Guppy 3.6.1 (https://community.nanoporetech.com/protocols/Guppy-protocol/v/gpb_2003_v1_revt_14dec2018) with the high-accuracy flip-flop algorithm (dna_r9.4.1_450bps_hac.cfg configuration file). Then, adapter sequences were removed using Porechop (https://github.com/rrwick/Porechop), and low-quality reads (average Q < 6) were filtered out using Trimmomatic 0.32 (Bolger et al., 2014). Illumina reads were also filtered (minimum read length—50) and trimmed (trailing, minimum Q—28) using the Trimmomatic tool.

Genome assemblies based on the Nanopore reads were performed using four assemblers: Canu 2.0 (Koren et al., 2017), Flye 2.7 (Kolmogorov et al., 2019), Shasta 0.5.0 (Shafin et al., 2020), and wtdbg2 2.5 (Ruan and Li, 2020). The default parameters were used, except for the minimum read length for Shasta (was set to 3,000 bp) and expected genome size for Flye and wtdbg2 (was set to 400 Mb). The statistics for the genome assemblies were calculated using QUAST 5.0.2 (Gurevich et al., 2013), and are presented in Table 1. Canu produced the longest assembly (361.7 Mb for contigs and 393.9 Mb for unitigs, which means high-confidence contigs) and largest contig of 5 Mb, and was one of the best in most parameters of Nx and Lx statistics. The highest N50 value of 365 kb was obtained using wtdbg2; however, the total assembly length was only 212.2 Mb, almost less than twice the real size of the flax genome (Wang et al., 2012). Canu was the second in N50 value, resulting in 350 kb for contigs and 225 kb for unitigs.

**Table 1:** QUAST statistics for genome assemblies of flax cultivar Atlant.

**Feature**	**Canu 2.0 contigs**	**Canu 2.0 unitigs**	**Flye 2.7**	**Shasta 0.5.0**	**wtdbg2 2.5**
**Total assembly length, Mb**	361.7	393.9	346.1	290.8	212.2
Number of contigs	2458	4361	8278	5641	2306
Largest contig, Mb	5.0	1.7	3.3	2.0	3.0
GC, %	38.94	38.93	38.98	39.06	39.03
**N50, kb**	350	225	191	295	365
NG50, kb	412	286	222	264	117
N75, kb	154	84	51	142	112
NG75, kb	220	159	84	106	–
**L50**	261	472	368	278	141
LG50	201	319	295	323	397
L75	648	1191	1205	634	407
LG75	463	685	871	789	–

*Key parameters are marked in bold. NG50/NG75 is the maximum length for which the subset of contigs of that length or longer covers at least 50%/75% of the reference genome (cultivar CDC Bethune, GenBank: GCA_000224295.2). LG50/LG75 is the number of contigs with a length equal to or greater than NG50/NG75, that is, the minimal number of contigs that cover 50%/75% of the reference genome. Unitigs are high-confidence contigs, according to Canu terminology*.

The misassembly rates between our assemblies and the NCBI representative genome for *L. usitatissimum* (cultivar CDC Bethune, GenBank: GCA_000224295.2) were evaluated using QUAST (Supplementary Data 1). Canu resulted in the best coverage of the reference genome (~95% for both contigs and unitigs) and the largest alignment (662 kb for both contigs and unitigs) and lost only to Shasta in one of the key parameters, NA50, which is an analog of N50 for fragments successfully mapped to the reference. Considering the rate of misassemblies larger than 1 kb and duplication ratio, Canu was only third after Shasta and wtdbg2; however, the latter demonstrated very low coverage of the reference genome (only 63.25%). It should be taken into account that we compared Atlant assemblies with the genome of another cultivar; therefore, it is naturally that some under- and misassemblies are present. The aforementioned statistics allowed, firstly, a comparison of the current Atlant assemblies performed with different tools.

**Figure 1:**
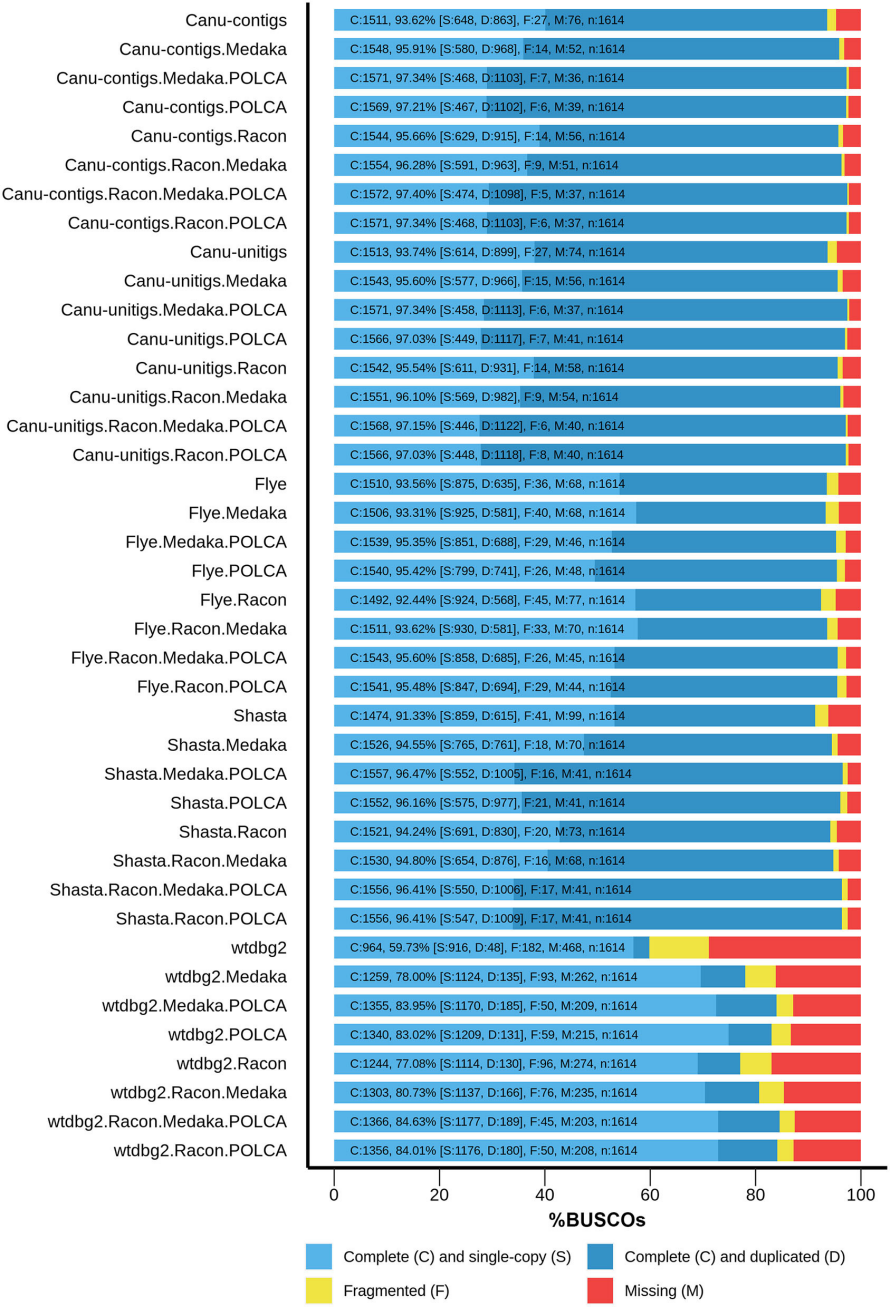
BUSCO assessment results for genome assemblies of flax cultivar Atlant. Results for the following assemblers are presented: Canu 2.0 contigs, Canu 2.0 unitigs, Flye 2.7, Shasta 0.5.0, and wtdbg2 2.5 coupled with Racon 1.4.3, Medaka 1.0.3, and/or POLCA from MaSuRCA 3.3.9 polishers.

Thereafter, the assemblies were polished, using Nanopore reads, with Racon 1.4.3 (Vaser et al., 2017) and/or Medaka 1.0.3 (https://github.com/nanoporetech/medaka), and, using Illumina reads, with the POLCA tool from the MaSuRCA 3.3.9 assembler (Zimin et al., 2017) to improve the contig accuracy. The assembly completeness was evaluated as the content of universal single-copy genes inherent to land plants using BUSCO v4, embryophyta_odb10 dataset (Seppey et al., 2019). The results are presented in Figure 1. For assemblies before polishing, the best results were obtained for Canu unitigs (93.74%), Canu contigs (93.62%), and Flye contigs (93.56%), whereas the worst result was shown for contigs assembled by wtdbg2 (59.73%). The highest efficiency of polishing was peculiar to the combination of Racon + Medaka + POLCA, which improved the completeness of the assembly from 93.62 to 97.40% (Canu contigs). This result was the best among those of all variants of assembler–polisher combinations. The totality of the parameters, including Nx and BUSCO statistics, as well as the misassemblies, suggested that the Canu genome assembly of flax cultivar Atlant polished according to Racon + Medaka + POLCA scheme was best, and it was used for further genome annotation.

The large percentage of duplicated BUSCOs (68% for the polished Canu assembly) is noteworthy. This is in good agreement with the statement that *L. usitatissimum* originated as the result of the hybridization of two diploid *Linum* species, from each of which it received a whole set of chromosomes (Bolsheva et al., 2017).

In the NCBI genome database, assemblies of only three *L. usitatissimum* genomes are presented: linseed cultivar CDC Bethune (representative genome, chromosome level, GenBank: GCA_000224295.2), linseed cultivar Longya-10 (scaffold level, GenBank: GCA_010665275.1), and fiber flax cultivar Heiya-14 (scaffold level, GenBank: GCA_010665265.1). For all three genomes, annotations have not been submitted that complicates the use of these data in studies of flax. In the present study, we annotated the assembled genome of fiber flax cultivar Atlant using the funannotate 1.8.0 pipeline (https://funannotate.readthedocs.io/en/latest/). Immediately before the annotation, repeat masking was performed with TANTAN (http://cbrc3.cbrc.jp/~martin/tantan/). Approximately 7.6% of the genomic sequence was masked. For the annotation, we used our previously obtained transcriptome sequencing data for five different tissues of cultivar Atlant (NCBI SRA: SRX8380594—shoots of seedlings, SRX8380593—roots of seedlings, SRX8380592—flowers of adult plants, SRX8380591—stems of adult plants, and SRX8380590—leaves of adult plants). To make genome-guided transcriptome assembly, we mapped the RNA-Seq reads to the assembled genome *via* HISAT2 2.2.0 (Kim et al., 2019). About 96% of reads (54.0M of 56.2M) were successfully mapped. 82,290 transcripts corresponding to 69,143 genomic loci were assembled using Trinity 2.8.5 in genome-guided mode. Based on the transcript data and mapped RNA-Seq reads, a total of 77,522 gene models were predicted using PASA 2.4.1, Augustus 3.3.3, GlimmerHMM 3.0.4, SNAP v. 2006-07-28, GeneMark 4.61, and CodingQuarry 2.0 (the results were combined and analyzed using EvidenceModeller 1.1.1). Among them, 1,182 were referred to as tRNA. In total, 18,946 gene models were successfully annotated using the Pfam database (up-to-date on June 2020), 19,741 using eggNOG (up-to-date on June 2020), 953 using BUSCO embryophyta_odb10 dataset, and 3,725 using UniProt (up-to-date on June 2020). The summary statistics of the functional annotation of predicted genes are presented in Supplementary Data 2. The assembled genome was deposited in the NCBI database under the BioProject accession number PRJNA648016.

## Conclusions

In this study, the genome of fiber flax cultivar Atlant was sequenced for the first time, using both Oxford Nanopore and Illumina platforms. For successful Nanopore sequencing, a protocol for extraction of pure high-molecular-weight DNA from the leaves of a single flax plant was developed. Sequencing of this DNA on the ONT platform resulted in 23 × flax genome coverage (8.4 Gb, N50 = 12 kb). On the Illumina platform, 30 × genome coverage was obtained (22.6 million of 250 + 250 paired-end reads). Genome assemblies were performed using Canu, Flye, Shasta, and wtdbg2. Subsequent polishing by Racon, Medaka, and POLCA was used to improve the contig accuracy. The most complete and accurate assembly was achieved by Canu with the polishing scheme Racon + Medaka + POLCA: total length = 361.7 Mb, N50 = 350 kb, and 97.40% completeness according to BUSCO. The genome was annotated using the funannotate pipeline and our transcriptome sequencing data for 5 different tissues of cultivar Atlant. The obtained results are useful for the evaluation of *L. usitatissimum* polymorphism at the genome level, the identification of sequences specific to fiber flax, as a reference in studies of fiber flax cultivars, and the development of flax genomic selection and genome editing. These findings can also be used for the analysis of flax DNA methylation at the whole-genome level, as information on this DNA modification can be derived from Nanopore reads.

## Data Availability Statement

The raw sequencing data and the assembled genome are deposited in the NCBI database under the BioProject accession number PRJNA648016.

## Author Contributions

AD, TR, and NM conceived and designed the work. EP, RN, AB, TR, NB, LP, ED, PK, AS, and NM performed the experiments. AD, EP, TR, AZ, OM, AK, GK, and NM analyzed the data. AD, EP, TR, GK, and NM wrote the manuscript. All authors read and approved the final manuscript.

## Conflict of Interest

The authors declare that the research was conducted in the absence of any commercial or financial relationships that could be construed as a potential conflict of interest.

## References

[B1] Ali E. Saand M. A. Khan A. R. Shah J. M. Feng S. Ming C.. (2020). Genome-wide identification and expression analysis of detoxification efflux carriers (DTX) genes family under abiotic stresses in flax. Physiol. Plant. 10.1111/ppl.13105. [Epub ahead of print]. 32270877

[B2] Atton M. (1989). Flax Culture: From Flower to Fabric. Owen Sound, ON: Ginger Press.

[B3] Belser C. Istace B. Denis E. Dubarry M. Baurens F. C. Falentin C.. (2018). Chromosome-scale assemblies of plant genomes using nanopore long reads and optical maps. Nat. Plants 4, 879–887. 10.1038/s41477-018-0289-4 30390080

[B4] Bolger A. M. Lohse M. Usadel B. (2014). Trimmomatic: a flexible trimmer for Illumina sequence data. Bioinformatics 30, 2114–2120. 10.1093/bioinformatics/btu170 24695404 PMC4103590

[B5] Bolsheva N. L. Melnikova N. V. Kirov I. V. Dmitriev A. A. Krasnov G. S. Amosova capital A.. (2019). Characterization of repeated DNA sequences in genomes of blue-flowered flax. BMC Evol. Biol. 19:49. 10.1186/s12862-019-1375-6 30813893 PMC6391757

[B6] Bolsheva N. L. Melnikova N. V. Kirov I. V. Speranskaya A. S. Krinitsina A. A. Dmitriev A. A.. (2017). Evolution of blue-flowered species of genus linum based on high-throughput sequencing of ribosomal RNA genes. BMC Evol. Biol. 17:253. 10.1186/s12862-017-1105-x 29297314 PMC5751768

[B7] Caligiuri S. P. Edel A. L. Aliani M. Pierce G. N. (2014). Flaxseed for hypertension: implications for blood pressure regulation. Curr. Hypertens. Rep. 16:499. 10.1007/s11906-014-0499-8 25342643

[B8] Campos J. R. Severino P. Ferreira C. S. Zielinska A. Santini A. Souto S. B.. (2019). Linseed essential oil–source of lipids as active ingredients for pharmaceuticals and nutraceuticals. Curr. Med. Chem. 26, 4537–4558. 10.2174/0929867325666181031105603 30378485

[B9] Corino C. Rossi R. Cannata S. Ratti S. (2014). Effect of dietary linseed on the nutritional value and quality of pork and pork products: systematic review and meta-analysis. Meat. Sci. 98, 679–688. 10.1016/j.meatsci.2014.06.041 25089794

[B10] Dhakal H. N. Sain M. (2019). Enhancement of mechanical properties of flax-epoxy composite with carbon fibre hybridisation for lightweight applications. Materials 13:109. 10.3390/ma13010109 31881745 PMC6981686

[B11] Diederichsen A. Richards K. (2003). “Cultivated flax and the genus linum L.: taxonomy and germplasm conservation,” in Flax: The Genus Linum, eds A. D. Muir and N. D. Westcott (London: CRC Press), 22–54.

[B12] Dmitriev A. A. Kezimana P. Rozhmina T. A. Zhuchenko A. A. Povkhova L. V. Pushkova E. N.. (2020a). Genetic diversity of SAD and FAD genes responsible for the fatty acid composition in flax cultivars and lines. BMC Plant Biol. 20:301. 10.1186/s12870-020-02499-w 33050879 PMC7557025

[B13] Dmitriev A. A. Krasnov G. S. Rozhmina T. A. Zyablitsin A. V. Snezhkina A. V. Fedorova M. S.. (2019). Flax (*Linum usitatissimum* L.) response to non-optimal soil acidity and zinc deficiency. BMC Plant Biol. 19:54. 10.1186/s12870-019-1641-1 30813909 PMC6393972

[B14] Dmitriev A. A. Novakovskiy R. O. Pushkova E. N. Rozhmina T. A. Zhuchenko A. A. Bolsheva N. L.. (2020b). Transcriptomes of different tissues of flax (*Linum usitatissimum* L.) cultivars with diverse characteristics. Front. Genet. 11:565146. 10.3389/fgene.2020.565146 33363567 PMC7755106

[B15] Fombuena V. Petrucci R. Dominici F. Jorda-Vilaplana A. Montanes N. Torre L. (2019). Maleinized linseed oil as epoxy resin hardener for composites with high bio content obtained from linen byproducts. Polymers 11:301. 10.3390/polym11020301 30960285 PMC6419190

[B16] Goodwin S. McPherson J. D. McCombie W. R. (2016). Coming of age: ten years of next-generation sequencing technologies. Nat. Rev. Genet. 17, 333–351. 10.1038/nrg.2016.49 27184599 PMC10373632

[B17] Goudenhooft C. Bourmaud A. Baley C. (2019). Flax (Linum usitatissimum L.) fibers for composite reinforcement: exploring the link between plant growth, cell walls development, and fiber properties. Front. Plant Sci. 10:411. 10.3389/fpls.2019.00411 31001310 PMC6456768

[B18] Goyal A. Sharma V. Upadhyay N. Gill S. Sihag M. (2014). Flax and flaxseed oil: an ancient medicine & modern functional food. J. Food Sci. Technol. 51, 1633–1653. 10.1007/s13197-013-1247-9 25190822 PMC4152533

[B19] Guo D. Jiang H. Yan W. Yang L. Ye J. Wang Y.. (2019). Resequencing 200 flax cultivated accessions identifies candidate genes related to seed size and weight and reveals signatures of artificial selection. Front. Plant Sci. 10:1682. 10.3389/fpls.2019.01682 32010166 PMC6976528

[B20] Gurevich A. Saveliev V. Vyahhi N. Tesler G. (2013). QUAST: quality assessment tool for genome assemblies. Bioinformatics 29, 1072–1075. 10.1093/bioinformatics/btt086 23422339 PMC3624806

[B21] Kezimana P. Dmitriev A. A. Kudryavtseva A. V. Romanova E. V. Melnikova N. V. (2018). Secoisolariciresinol diglucoside of flaxseed and its metabolites: biosynthesis and potential for nutraceuticals. Front. Genet. 9:641. 10.3389/fgene.2018.00641 30619466 PMC6299007

[B22] Kim D. Paggi J. M. Park C. Bennett C. Salzberg S. L. (2019). Graph-based genome alignment and genotyping with HISAT2 and HISAT-genotype. Nat. Biotechnol. 37, 907–915. 10.1038/s41587-019-0201-4 31375807 PMC7605509

[B23] Kolmogorov M. Yuan J. Lin Y. Pevzner P. A. (2019). Assembly of long, error-prone reads using repeat graphs. Nat. Biotechnol. 37, 540–546. 10.1038/s41587-019-0072-8 30936562

[B24] Koren S. Walenz B. P. Berlin K. Miller J. R. Bergman N. H. Phillippy A. M. (2017). Canu: scalable and accurate long-read assembly via adaptive k-mer weighting and repeat separation. Genome Res. 27, 722–736. 10.1101/gr.215087.116 28298431 PMC5411767

[B25] Li C. Lin F. An D. Wang W. Huang R. (2017). Genome sequencing and assembly by long reads in plants. Genes 9:6 10.3390/genes9010006 PMC5793159 29283420

[B26] Li F. W. Harkess A. (2018). A guide to sequence your favorite plant genomes. Appl. Plant Sci. 6:e1030. 10.1002/aps3.1030 29732260 PMC5895188

[B27] Mokhothu T. H. John M. J. (2015). Review on hygroscopic aging of cellulose fibres and their biocomposites. Carbohydr. Polym. 131, 337–354. 10.1016/j.carbpol.2015.06.027 26256193

[B28] Morello L. Pydiura N. Galinousky D. Blume Y. Breviario D. (2020). Flax tubulin and CesA superfamilies represent attractive and challenging targets for a variety of genome- and base-editing applications. Funct. Integr. Genomics 20, 163–176. 10.1007/s10142-019-00667-2 30826923

[B29] Muir A. D. Westcott N. D. (2003). Flax: The Genus Linum. London: CRC Press.

[B30] Novakovskiy R. O. Povkhova L. V. Krasnov G. S. Rozhmina T. A. Zhuchenko A. A. Kudryavtseva L. P. (2019). The cinnamyl alcohol dehydrogenase gene family is involved in the response to Fusarium oxysporum in resistant and susceptible flax genotypes. Vavilov J. Genet. Breed. 23, 896–901. 10.18699/VJ19.564

[B31] Parikh M. Maddaford T. G. Austria J. A. Aliani M. Netticadan T. Pierce G. N. (2019). Dietary flaxseed as a strategy for improving human health. Nutrients 11:1171. 10.3390/nu11051171 31130604 PMC6567199

[B32] Rozhmina T. A. Zhuchenko A. A. Melnikova N. V. Smirnova A. D. (2020). Resistance of flax gene pool samples to edaphic stress caused by low acidity. Agric. Sci. Euro North East. 21, 133–140. 10.30766/2072-9081.2020.21.2.133-140

[B33] Ruan J. Li H. (2020). Fast and accurate long-read assembly with wtdbg2. Nat. Methods 17, 155–158. 10.1038/s41592-019-0669-3 31819265 PMC7004874

[B34] Ryzhov A. I. Rozhmina T. A. Golubeva L. M. (2012). The role of the flax gene pool in obtaining competitive fiber products. Technol. 21st Century Light Ind. 6, 1–11.

[B35] Saha D. Mukherjee P. Dutta S. Meena K. Sarkar S. K. Mandal A. B.. (2019). Genomic insights into HSFs as candidate genes for high-temperature stress adaptation and gene editing with minimal off-target effects in flax. Sci. Rep. 9:5581. 10.1038/s41598-019-41936-1 30944362 PMC6447620

[B36] Seppey M. Manni M. Zdobnov E. M. (2019). BUSCO: assessing genome assembly and annotation completeness. Methods Mol. Biol. 1962, 227–245. 10.1007/978-1-4939-9173-0_14 31020564

[B37] Shafin K. Pesout T. Lorig-Roach R. Haukness M. Olsen H. E. Bosworth C.. (2020). Nanopore sequencing and the Shasta toolkit enable efficient de novo assembly of eleven human genomes. Nat. Biotechnol. 38, 1044–1053. 10.1038/s41587-020-0503-6 32686750 PMC7483855

[B38] Singh K. K. Mridula D. Rehal J. Barnwal P. (2011). Flaxseed: a potential source of food, feed and fiber. Crit. Rev. Food Sci. Nutr. 51, 210–22. 10.1080/10408390903537241 21390942

[B39] Song J. M. Guan Z. Hu J. Guo C. Yang Z. Wang S.. (2020). Eight high-quality genomes reveal pan-genome architecture and ecotype differentiation of Brassica napus. Nat. Plants 6, 34–45. 10.1038/s41477-019-0577-7 31932676 PMC6965005

[B40] Vaisey-Genser M. Morris D. H. (2003). “Introduction: History of the Cultivation and Uses of Flaxseed,” in Flax: The Genus Linum, eds A. D. Muir and N. D. Westcott (London: CRC Press), 1–21.

[B41] Vaser R. Sovic I. Nagarajan N. Sikic M. (2017). Fast and accurate *de novo* genome assembly from long uncorrected reads. Genome Res. 27, 737–746. 10.1101/gr.214270.116 28100585 PMC5411768

[B42] Wang Z. Hobson N. Galindo L. Zhu S. Shi D. McDill J.. (2012). The genome of flax (*Linum usitatissimum*) assembled de novo from short shotgun sequence reads. Plant J. 72, 461–473. 10.1111/j.1365-313X.2012.05093.x 22757964

[B43] Wu C. M. Lai W. Y. Wang C. Y. (2016). Effects of surface modification on the mechanical properties of flax/beta-polypropylene composites. Materials 9:314. 10.3390/ma9050314 28773439 PMC5503065

[B44] Wu W. Nemri A. Blackman L. M. Catanzariti A. M. Sperschneider J. Lawrence G. J.. (2019). Flax rust infection transcriptomics reveals a transcriptional profile that may be indicative for rust Avr genes. PLoS ONE 14:e0226106. 10.1371/journal.pone.0226106 31830116 PMC6907798

[B45] You F. M. Cloutier S. (2020). Mapping quantitative trait loci onto chromosome-scale pseudomolecules in flax. Methods Protoc. 3:28. 10.3390/mps3020028 32260372 PMC7359702

[B46] You F. M. Xiao J. Li P. Yao Z. Jia G. He L.. (2018). Chromosome-scale pseudomolecules refined by optical, physical and genetic maps in flax. Plant J. 95, 371–384. 10.1111/tpj.13944 29681136

[B47] Zhang B. Zhu W. Diao S. Wu X. Lu J. Ding C.. (2019). The poplar pangenome provides insights into the evolutionary history of the genus. Commun. Biol. 2:215. 10.1038/s42003-019-0474-7 31240253 PMC6581948

[B48] Zhang H. Liu D. Huang T. Hu Q. Lammer H. (2020a). Three-dimensional printing of continuous flax fiber-reinforced thermoplastic composites by five-axis machine. Materials 13:1678. 10.3390/ma13071678 32260222 PMC7178657

[B49] Zhang J. Qi Y. Wang L. Wang L. Yan X. Dang Z.. (2020b). Genomic comparison and population diversity analysis provide insights into the domestication and improvement of flax. iScience 23:100967. 10.1016/j.isci.2020.100967 32240956 PMC7114909

[B50] Zhu J. Zhu H. Njuguna J. Abhyankar H. (2013). Recent development of flax fibres and their reinforced composites based on different polymeric matrices. Materials 6, 5171–5198. 10.3390/ma6115171 28788383 PMC5452774

[B51] Zimin A. V. Puiu D. Luo M. C. Zhu T. Koren S. Marcais G.. (2017). Hybrid assembly of the large and highly repetitive genome of *Aegilops tauschii*, a progenitor of bread wheat, with the MaSuRCA mega-reads algorithm. Genome Res. 27, 787–792. 10.1101/gr.213405.116 28130360 PMC5411773

