# Supplementary material for: Genome Sequencing of Fiber Flax Cultivar Atlant Using Oxford Nanopore and Illumina Platforms
Source: Front Genet. 2021 Jan 14;11:590282. doi: 10.3389/fgene.2020.590282 (PMC7841463; doi:10.3389/fgene.2020.590282)
Supplement: Supplementary file 1 [file Data_Sheet_1.PDF]

Supplementary Data 1. Consistency between obtained genome assemblies of flax cultivar Atlant and NCBI representative genome of *Linum usitatissimum* (cultivar CDC Bethune, GenBank: GCA_000224295.2).

**Feature**	**Canu 2.0 contigs**	**Canu 2.0 unitigs**	**Flye 2.7**	**Shasta 0.5.0**	**wtdbg2 2.5**
**Covered reference genome fraction, %**	94.76	94.87	88.61	87.89	63.25
Duplication ratio	1.10	1.18	1.21	1.05	1.05
**Misassemblies (large: > 1 kb)**	11 547	12 125	13 391	9 096	10 640
Misassembled contigs	1202	1932	1906	1295	1103
Misassembled contigs length, Mb	291.2	297.4	259.9	247.6	184.9
**Local misassemblies (small: < 1 kb)**	45 893	54 578	62 248	36 503	37 568
Unaligned contigs – completely	339	471	457	1 845	129
Unaligned contigs – partially	1 964	3 475	4 444	2 918	1 708
Unaligned length, Mb	80.6	92.7	57.3	42.7	33.2
Mismatches per 100 kbp	1024	1225	1535	870	1465
Indels per 100 kbp	381	462	577	332	546
Largest alignment, kb	662	662	393	554	316
Total aligned length, Mb	279.6	299.5	286.0	246.5	177.5
NA50, kb	49	40	26	58	28
NGA50, kb	62	58	30	51	8
NA75, kb	1	0	6	18	8
NGA75, kb	21	24	11	4	-
LA50	1 792	2 269	3 199	1 369	1 917
LGA50	1 376	1 466	2 654	1 602	5 152
LA75	9 223	23 086	9 628	3 501	5 284
LGA75	3 499	3 548	6 984	5 674	-

worse	***mid***	***better***

Note: The color scale indicates the quality of parameter value from worse (pink) to better (green). Key parameters are marked in bold. NG50/NG75 is the maximum length for which the subset of contigs of that length or longer covers at least 50%/75% of the reference genome (cultivar CDC Bethune, GenBank: GCA_000224295.2). LG50/LG75 is the number of contigs with a length equal to or greater than NG50/NG75, that is, the minimal number of contigs that cover 50%/75% of the reference genome. NA50/75, NGA50/75, LA50/75, LGA50/75 (“A” stands for “aligned”) are similar to the corresponding metrics without “A”, but in this case, aligned blocks instead of contigs are considered. Unitigs are high-confidence contigs, according to Canu terminology.
